# Influence of Mild Thyroid Dysfunction on Outcomes after Off-Pump Coronary Artery Bypass Surgery

**DOI:** 10.3390/jcm11175033

**Published:** 2022-08-27

**Authors:** Young-Eun Joe, Yu Rim Shin, Young-Lan Kwak, Jae Hang Shim, Young Suk Shon, Jae-Kwang Shim

**Affiliations:** 1 Department of Anesthesiology and Pain Medicine, Anesthesia and Pain Research Institute, Yonsei University College of Medicine, 50-1 Yonsei-ro, Seodaemun-gu, Seoul 03722, Korea; 2 Department of Thoracic and Cardiovascular Surgery, Yonsei University College of Medicine, Seoul 03722, Korea; 3 Department of Anesthesiology and Pain Medicine, Hanyang University Guri Hospital, Guri-si 11923, Gyeonggi-do, Korea

**Keywords:** mild thyroid dysfunction, subclinical hypothyroidism, low T3, coronary artery bypass, off-pump

## Abstract

We retrospectively evaluated the association between preoperative mild thyroid dysfunction (subclinical hypothyroidism [SCH] or low triiodothyronine [T3] syndrome) and outcomes in patients who underwent off-pump coronary surgery (OPCAB). Further, 800 patients (2015–2020) were divided into euthyroid, low T3, and SCH groups. The primary outcome assessed the association with composite endpoints (myocardial infarction, prolonged mechanical ventilation [>24 h], acute kidney injury, and 30-day/in-hospital mortality). The secondary outcome assessed the association with long-term mortality and 10% and 8% of the patients exhibited low T3 and SCH, respectively. Incidences of composite endpoints were significantly higher in the low T3 and SCH groups versus the euthyroid group (50.6%, 45.2%, 17.4%, respectively, *p* < 0.001). Multivariable regression analysis revealed chronic kidney disease, anemia, EuroSCORE, low T3, and SCH as independent risk factors of composite endpoints. The long-term mortality rate (median follow-up, 30 months) was higher in the low T3 and SCH groups than in the euthyroid group (9.6%, 11.3%, 2.4%, respectively, *p* < 0.001). In the absence of overt thyroid dysfunction, low T3 and SCH were associated with increased risk of adverse outcomes after OPCAB. Moreover, the adverse influences of low T3 and SCH seem to extend to long-term mortality, implying that routine thyroid function tests may enhance accurate risk stratification.

## 1. Introduction

Patients undergoing coronary artery bypass graft surgery (CABG) comprise a high-risk population; thus, consistent efforts have been made to identify the risk factors for precise risk stratification and improve postsurgical outcomes [1,2]. In that context, the influence of thyroid hormones on the cardiovascular system, as well as on cardiac function in disease states, such as remodeling after myocardial infarction (MI), has long been recognized [3,4,5]. Of interest, patients with cardiac diseases have frequently exhibited mild thyroid dysfunction in the form of subclinical hypothyroidism (SCH) or low triiodothyronine (T3) syndrome without any apparent pathologies involving the thyroid gland [6,7]. The close associations between SCH or low T3 syndrome and cardiac disease mortality were more prominent in patients with ischemic heart disease [8,9]. Similar results were observed in patients who underwent percutaneous coronary intervention (PCI), including patients with acute MI [10,11,12]. In the surgical population, relevant evidence is scarce, and the prognostic importance of low T3 syndrome or SCH on mortality had only been revealed in patients undergoing on-pump CABG [13,14].

Patients who undergo off-pump CABG (OPCAB) share common risk factors with PCI or on-pump CABG patients. However, by evading cardiopulmonary bypass (CPB), OPCAB presents different risk profiles than its on-pump counterpart or PCI [15,16]. Thus, the influence of mild thyroid dysfunction, which can easily be identified with a thyroid function test (TFT), on the outcomes after OPCAB may be different, while no comprehensive evidence exists. In addition, finding such a relationship is of high priority, as thyroid hormone therapy in selected patients may yield improved outcomes. 

Therefore, this retrospective analysis aimed to evaluate the association between SCH or low T3 syndrome with the outcomes in patients who underwent OPCAB.

## 2. Materials and Methods

### 2.1. Patient 

**Figure 1:**
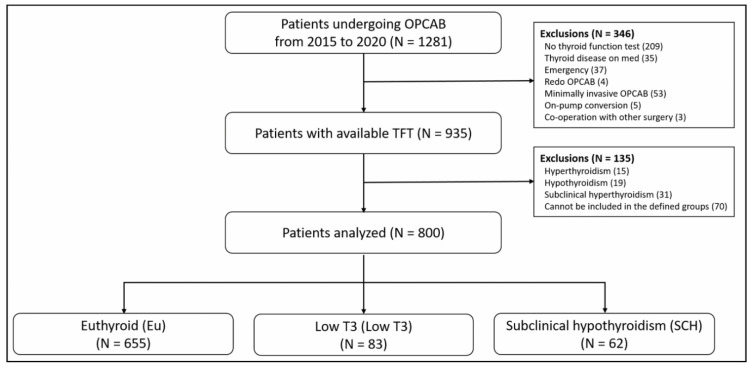
Flow chart of patients’ enrollment. OPCAB (off-pump coronary artery bypass graft); SCH (subclinical hypothyroidism); TFT (thyroid function test); T3 (triiodothyronine).

This retrospective study was approved by the Institutional Review Board of the Yonsei University Health System (4-2021-0587, 2021-06-21). The requirement for patient consent was exempted. Overall, 1281 patients who underwent OPCAB at the Severance Cardiovascular Hospital from 2015 to 2020 were enrolled in the study (at our institution, the first option for isolated CABG is OPCAB); 346 patients were excluded due to lack of preoperative TFT results, diagnosis of thyroid disease that requires medication, emergency conditions, redo OPCAB, minimally invasive OPCAB, on-pump conversion, and co-operation with other surgeries (Figure 1). Of the remaining 935 patients, 135 patients were further excluded due to the following pre-operative TFT results: 19 patients with hypothyroidism, 15 patients with hyperthyroidism, 31 patients with subclinical hyperthyroidism, and 70 patients with atypical thyroid status, which could not be clustered in any of the pre-defined groups.

### 2.2. Thyroid Function Test and Patient Group Allocation 

Serum thyroid hormone concentrations were measured using the immunoassay analyzer (ARCHITECT i2000, Abbott core laboratory, Abbott Park, IL, USA). The reference values for triiodothyronine (T3), thyroxine (T4), and thyroid-stimulating hormone (TSH) were 0.61–1.6 ng/mL, 0.8–1.23 ng/dL, and 0.41–4.31 µIU/mL, respectively. TFT was performed in the morning or 1 day before surgery in 79% of the patients (1 [1, 2] days). A total of 655 (82%) patients with normal TFT results were designated to the euthyroid group, 83 (10%) patients with T3 levels below the reference range but normal TSH and free T4 levels were designated to the low T3 group; and 62 (8%) patients with TSH levels above the reference range, but with normal T3 and free T4 levels, were designated to the SCH group. 

### 2.3. Anesthetic Management and Surgical Procedure

All operations were performed by two surgical teams with median sternotomy. Institutional standardized anesthetic and surgical management were provided to all the patients [17]. Standard monitoring included the use of a Swan-Ganz catheter and transesophageal echocardiography. Anesthesia was maintained with 0.4–1.5% sevoflurane and continuous infusion of sufentanil (0.3–0.5 μg·kg^−1^·h^−1^). Major hemodynamic goals during surgery were to maintain the mean arterial pressure above 70 mm Hg and mixed venous oxygen saturation above 60%.

### 2.4. Study Endpoints and Variables

The primary endpoint was to assess the association of low T3 and SCH with the composite of morbidity endpoints, including postoperative MI [18] (within 48 h after surgery), prolonged mechanical ventilation (>24 h), acute kidney injury [19] (within 7 days after surgery), and 30-day or in-hospital mortality. Postoperative MI was defined as type 5 MI using the fourth universal definition of MI [18]. Acute kidney injury was defined as “Kidney Disease: Improving Global Outcomes” stage 1 or higher [19]. These commonly assessed complications after cardiac surgery were chosen for their close association with cardiovascular and endothelial functions, which are influenced by thyroid hormones. The secondary endpoint evaluated the association of low T3 and SCH with long-term, all-cause mortality, except cancer or accidents.

Regarding preoperative risk factors, anemia was defined as a hemoglobin level of ≤13 g/dL for men and ≤12 g/dL for women, and hypoalbuminemia as a serum albumin level of ≤3.5 g/dL. Congestive heart failure was defined based on New York Heart Association class ≥ III. Acute coronary syndrome was defined as non-ST-elevation MI or unstable angina before surgery. Recent MI was defined as the occurrence of MI within 3 months and left main disease as >70% luminal stenosis by coronary angiography. 

### 2.5. Statistical Analysis

Continuous variables are presented as mean ± standard deviation or as median [interquartile range] and categorical variables as numbers (percentages). Inter-group comparisons of continuous variables were performed using ANOVA or the Kruskal–Wallis test, using the post-hoc analysis of the Dwass–Steel–Critchlow–Fligner method for multiple comparisons. Categorical variables were compared using chi-square (χ^2^) or Fisher’s exact tests using the post-hoc analysis of Bonferroni’s adjustment. 

Univariable and multivariable logistic regression analysis was performed to find independent risk factors of the primary endpoint. The following variables were selected a priori and introduced in the logistic regression analysis to minimize the introduction of selection bias: age, sex, body mass index (BMI), hypertension, atrial fibrillation, chronic kidney disease (CKD), cerebrovascular accident, diabetes mellitus, chronic obstructive pulmonary disease, peripheral arterial occlusive disease (PAOD), congestive heart failure, recent MI, acute coronary syndrome, left main disease, hypoalbuminemia, anemia, and EuroSCORE. Multi-collinearity was checked by using the tolerance test and variance inflation factors. The long-term cumulative survival was estimated by the Kaplan–Meier survival curves followed by log-rank tests. Cox regression analysis was performed to identify independent risk factors of long-term mortality by adjusting for EuroSCORE. All statistical analyses were performed using SPSS version 26.0 (IBM Corp.; Armonk, NY, USA). A *p*-value of <0.05 was considered statistically significant.

## 3. Results

### 3.1. Patients’ Characteristics and Intraoperative Data

Baseline characteristics of the patients among the three groups according to the TFT results are shown in Table 1. The number of female patients and EuroSCORE were significantly higher in the low T3 group as compared to the other two groups, while the BMI and left ventricular ejection fraction were significantly lower. Diabetes mellitus and PAOD were significantly more prevalent in the low T3 group as compared to the other two groups. CKD was most prevalent in the low T3 group, followed by the SCH group, and was least prevalent in the euthyroid group. The difference in the prevalence of CKD was statistically significant for the three groups (54.2% vs. 27.4% vs. 9.3%, *p* < 0.001, respectively). Atrial fibrillation, congestive heart failure, and acute coronary syndrome were also significantly prevalent in the low T3 and SCH groups as compared to the euthyroid group. 

Table 2 represents preoperative and intraoperative data for the three groups. Creatinine and albumin levels were significantly different among the three groups, with albumin levels being the lowest and creatinine levels being the highest in the low T3 group, followed by the SCH group and, thirdly, the euthyroid group. The prevalence of anemia was also significantly different among the three groups, with the highest prevalence in the low T3 group, followed by the SCH group and, thirdly, the euthyroid group. Creatinine kinase-MB and Troponin T levels were significantly higher in the low T3 group compared with the other two groups. The number of grafts performed was similar among the three groups.

### 3.2. Analyses of the Primary and Secondary Endpoints 

The postoperative outcome data in Table 3 shows that the composite endpoint was significantly more prevalent in the low T3 and SCH groups compared to the euthyroid group. The 30-day in-hospital mortality rate was significantly higher in the low T3 group compared to the other two groups, while acute kidney injury was significantly more prevalent in the low T3 and SCH groups in comparison to the euthyroid group. The percentage of patients requiring prolonged mechanical ventilation was significantly higher in the SCH group compared with the other two groups. Patients in the low T3 and SCH groups exhibited significantly longer ICU and hospital stay compared with the euthyroid group. The long-term, all-cause mortality (except from cancer or accidents) rate was significantly higher in the low T3 and SCH groups compared to the euthyroid group. None of the patients died of complications related to permanent stroke. 

In the multivariable analysis, CKD, anemia, EuroSCORE, low T3, and SCH were identified as independent risk factors (Table 4). There was no multicollinearity between all variables used in the analysis.

**Figure 2:**
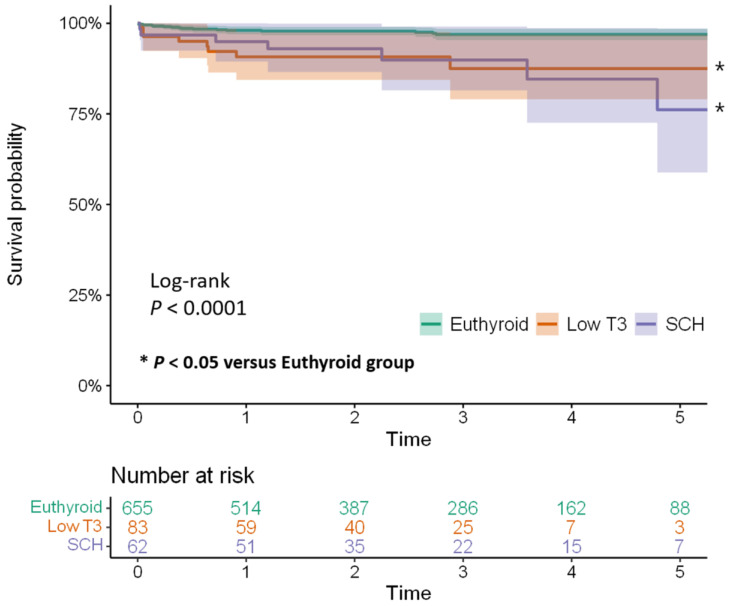
Kaplan–Meier curves for long-term, all-cause mortality (median follow-up of 30 months) with 95% confidence interval. * *p* < 0.05 versus Euthyroid group. SCH (subclinical hypothyroidism); T3 (triiodothyronine).

The Kaplan–Meier curves and the log-rank test results (Figure 2) show a significantly higher mortality rate in the low T3 and SCH groups compared to the euthyroid group (*p* < 0.001). Additionally, Cox regression analysis revealed low T3 and SCH independent risk factors for long-term mortality (Table 5).

## 4. Discussion

In this retrospective study, mild thyroid dysfunction in the form of low T3 (10%) or SCH (8%) was prevalent amongst patients who underwent isolated, multi-vessel OPCAB. Moreover, we identified a significant association between low T3 or SCH and detrimental outcomes of OPCAB. Although patients with low T3 or SCH had prevalent comorbidities, both remained independent risk factors for composite endpoint, including in-hospital/30-day mortality, even after adjusting for these confounders, showing a 2.3- and 2.8-fold increased risk, respectively. Furthermore, low T3 and SCH were significantly associated with long-term mortality (median follow-up, 30 months) when adjusted for EuroSCORE. 

Thyroid hormones bind to nuclear receptors and activate the cardiomyocyte ion channels [5] to regulate myocardial contractility and ventricular relaxation; they also boost cardiac output by decreasing systemic vascular resistance [5,7]. Moreover, thyroid hormones protect against myocardial ischemia and hinder the progression of unfavorable cardiac remodeling by reducing cell death and fibrous tissue deposition and improving myocardial perfusion [20,21,22]. Thyroid dysfunction also causes endothelial dysfunction, resulting in decreased synthesis of vasodilators and increased vascular permeability, aggravating kidney and lung damage [23]. Overt thyroid dysfunction is a harbinger of unfavorable prognosis in patients with heart disease [24,25,26]. Notably, mild subclinical thyroid dysfunction has been linked to pernicious outcomes in ischemic heart disease patients [4]. Only a few studies have demonstrated the prognostic importance of mild thyroid dysfunction related to adverse outcomes in patients who underwent on-pump CABG [13]; however, relevant evidence of this association is lacking in patients who underwent OPCAB. Notably, OPCAB exerts different influences on major morbidity endpoints compared to its on-pump counterpart by evading CPB [15], especially in terms of renal dysfunction, bleeding complications, and ventilation time [27]. Furthermore, CPB-induced hemodilution and inflammation would further cause alterations in intraoperative thyroid hormone concentrations, while these changes would be less in OPCAB, theoretically.

Our results indicate that mild thyroid dysfunction, in the form of low T3 or SCH, was independently associated with detrimental postoperative outcome, even when adjusted for well-known risk factors. Thus, it seems that OPCAB does not provide outcome benefit in patients with mild thyroid dysfunction, although direct comparisons with on-pump CABG were not possible. The composite of morbidity endpoints was mostly driven by acute kidney injury in the current study. Nonetheless, multivariable analysis showed that the SCH had the highest odds ratio (2.8), followed by CKD (2.7), and low T3 (2.3), suggesting the high prognostic importance of mild thyroid dysfunction. 

Although low T3 syndrome and SCH are forms of mild, subclinical thyroid dysfunction, they may be regarded as two different diseases. Low T3 syndrome in the absence of evident thyroid dysfunction mostly stems from decreased 5′-monodeiodinase activity, which subsequently reduces the peripheral conversion of free T4 to T3 [28,29]. This may occur in cardiac patients and situations related to systemic inflammation and acute disease states, such as starvation, sepsis, severe illness, and surgery [30]. Indeed, patients in the low T3 group had more comorbidities, such as PAOD, CKD, diabetes mellitus, and anemia, compared to the SCH group. Nevertheless, low T3 syndrome remained an independent risk factor of adverse outcomes when accounting for these risk factors. Apart from the protective effect of T3 against ischemia, T3 destitution, even for a short period of 4 days, has deleterious effects on myocardial histology, leading to structural and functional changes which may complicate the perioperative course [31]. 

Conversely, SCH seems more related to thyroid dysfunction. Thyroid hormone secretion is regulated through the hypothalamus–pituitary–thyroid gland axis via a negative feedback mechanism. Therefore, even if T3 and T4 are normal, high levels of TSH reflect inappropriate status of thyroid hormone regulation [32]. SCH is associated with endothelial dysfunction, coagulation abnormalities, and increased levels of C-reactive protein [26], which increases the risk of cardiovascular diseases, similar to overt hypothyroidism [4,33] and systemic inflammation as in low T3 syndrome [34]. This is consistent with our observation that CKD, atrial fibrillation, congestive heart failure, acute coronary syndrome, and anemia were more prevalent in the SCH group than in the euthyroid group. Irrespective of the underlying causes, the end-effect of low T3 syndrome and SCH on the cardiovascular system should be similar in reflecting mild thyroid dysfunction as observed for individual morbidity endpoints in the current study. Moreover, the adverse influence on long-term mortality was evident in both low T3 and SCH, which further strengthens the prognostic importance of mild thyroid dysfunction. Nevertheless, we analyzed low T3 and SCH as separate variables in the regression analysis, adjusting for major confounding variables (relevant co-morbidities), to provide comprehensive results accounting for their potentially different etiologies.

The current study firstly presents evidence regarding the prognostic importance of mild, subclinical thyroid dysfunction, in the form of low T3 syndrome and SCH, in a fairly large number of patients who underwent OPCAB, which has not been validated heretofore. As OPCAB clearly exerts different risk profiles than on-pump CABG, especially in terms of the primary endpoint morbidity variables (acute kidney injury, prolonged mechanical ventilation) that showed meaningful differences related to SCH or low T3 syndrome in the current study [27], our results provide novel evidence that would aid surgery-specific risk stratification. Despite being a potentially modifiable risk factor, thyroid hormone replacement before CABG was not beneficial in hemodynamic or outcome variables [35,36]. Yet, these studies targeted the influence of thyroid hormones on cardiac function after CPB, not accounting for the patients’ preoperative thyroid dysfunction. Moreover, concerns have been raised regarding the potential risk of myocardial ischemia and arrhythmia induced by thyroid hormone replacement and subsequent hyperthyroidism, especially in patients with limited coronary reserve. In contrast to the obvious need for thyroid hormone therapy in patients with overt hypothyroidism, the need for thyroid hormone therapy in patients with SCH or low T3 syndrome remains elusive. As our study showed adverse outcome in patients with mild thyroid dysfunction, it merits a further study on whether thyroid hormone replacement in these specific subsets of patients would actually result in improved prognosis. 

The current study has inherent limitations related to its retrospective nature. Further, although our primary endpoint was focused on the more relevant early outcome variables, TFT was not serially performed over the postoperative period, which may have confounded the long-term influence of low T3 syndrome or SCH on the long-term mortality (secondary endpoint). Also, although EuroSCORE encompasses many important variables, comprehensive adjustment for confounders of long-term mortality could not be performed to avoid overfitting the Cox regression analysis model (as opposed to the analysis for the primary endpoint).

## 5. Conclusions

Mild subclinical thyroid dysfunction, in low T3 syndrome or SCH, was associated with deleterious outcomes, including 30-day in-hospital mortality after OPCAB, along with well-known risk factors such as age, CKD, anemia, and EuroSCORE. Moreover, low T3 and SCH showed significant association with long-term mortality. Considering the frequent occurrence of mild thyroid dysfunction in CABG patients, TFT should form a routine part of the preoperative evaluation in this patient subset to facilitate accurate risk stratification.

## Figures and Tables

**Table 1:** Baseline characteristics of the patients among the three groups designated by thyroid function test.

	Euthyroid (N = 655)	Low T3 (N = 83)	SCH (N = 62)	*p*-Value
Age (years)	65.4 ± 9.36	66.5 ± 9.95	67.4 ± 8.31	0.122
Female	137 (20.9)	29 (34.9)	13 (21.0)	0.015 *
Body mass index (kg/m^2^)	24.5 [22.6, 26.6]	23.4 [21.1, 25.6]	24.4 [22.6, 25.6]	0.001 *
Hypertension	462 (70.5)	67 (80.7)	46 (74.2)	0.138
Diabetes mellitus	347 (53.0)	57 (68.7)	36 (58.1)	0.022 *
Chronic kidney disease	61 (9.3)	45 (54.2)	17 (27.4)	<0.001 *†‡
Old cerebral infarction	88 (13.4)	12 (14.5)	11 (17.7)	0.636
Atrial fibrillation	14 (2.1)	7 (8.4)	8 (12.9)	<0.001 *†
COPD	33 (5.0)	4 (4.8)	3 (4.8)	0.994
PAOD	29 (4.4)	9 (10.8)	3 (4.8)	0.044 *
Congestive heart failure	37 (5.6)	13 (15.7)	8 (12.9)	0.001 *†
Recent MI (<3 month)	132 (20.2)	23 (27.7)	16 (25.8)	0.193
Acute coronary syndrome	207 (31.6)	45 (54.2)	29 (46.8)	<0.001 *†
LVEF (%)	59 [46, 67]	45 [38, 57]	54 [40, 63]	<0.001 *
Left main disease	80 (12.2)	10 (12.0)	6 (9.7)	0.841
EuroSCORE	1.46 [0.87, 3.00]	2.21 [1.49, 3.98]	1.83 [1.05, 3.07]	<0.001 *
***Medications***				
Beta blocker	364 (55.6)	43 (51.8)	40 (64.5)	0.292
Calcium channel blocker	273 (41.7)	33 (39.8)	26 (41.9)	0.940
ACEI/ARB	375 (57.3)	44 (53.0)	32 (51.6)	0.560

Data are presented as mean ± SD, median [interquartile range], or n (%). * *p* < 0.05 Euthyroid vs. low T3, † *p* < 0.05 Euthyroid vs. SCH, ‡ *p* < 0.05 low T3 vs. SCH, representing post-hoc analysis. ACEI/ARB (angiotensin converting enzyme inhibitor/angiotensin receptor blocker); COPD (chronic pulmonary obstructive disease); EuroSCORE (European System for Cardiac Operative Risk Evaluation); LVEF (left ventricular ejection fraction); MI (myocardial infarction); PAOD (peripheral artery occlusive disease); SCH (subclinical hypothyroidism); T3 (triiodothyronine).

**Table 2:** Preoperative laboratory data and intraoperative data among the three groups, designated by thyroid function test.

	Euthyroid (N = 655)	Low T3 (N = 83)	SCH (N = 62)	*p*-Value
***Laboratory data***				
T3 (ng/mL)	0.84 [0.74, 0.93]	0.54 [0.45, 0.58]	0.77 [0.68, 0.92]	<0.001 *†‡
Free T4 (ng/dL)	0.96 [0.89, 1.04]	0.95 [0.88, 1.04]	0.94 [0.87, 1.01]	0.170
TSH (µIU/mL)	1.57 [1.03, 2.31]	1.53 [0.91, 2.15]	5.62 [4.82, 7.43]	<0.001 †‡
Creatinine (mg/dL)	0.87 [0.74, 1.04]	1.33 [0.78, 4.72]	0.95 [0.83, 1.34]	<0.001 *†‡
Albumin (g/dL)	4.1 [3.8, 4.4]	3.5 [3.3, 3.8]	3.8 [3.4, 4.2]	<0.001 *†‡
Anemia	252 (38.5)	69 (83.1)	41 (66.1)	<0.001 *†‡
Troponin T (pg/mL)	13.0 [8.0, 28.0]	91.0 [21.0, 414.0]	45.5 [13.0, 136.0]	<0.001 *‡
***Intraoperative data***				
Anesthetic time (min)	305 ± 41	299 ± 38	306 ± 37	0.402
Operation time (min)	235 ± 37	227 ± 35	233 ± 37	0.185
Number of grafts	3 [3, 4]	3 [3, 4]	3 [3, 4]	0.151

Data are presented as mean ± SD, median [interquartile range] or n (%). * *p* < 0.05 Euthyroid vs. low T3, † *p* < 0.05 Euthyroid vs. SCH, ‡ *p* < 0.05 low T3 vs. SCH, representing post-hoc analysis. CK (creatinine kinase); T4 (thyroxine); TSH (thyroid stimulating hormone); T3 (triiodothyronine).

**Table 3:** Postoperative data including primary and secondary outcomes among the three groups, designated by thyroid function test.

	Euthyroid (N = 655)	Low T3 (N = 83)	SCH (N = 62)	*p*-Value
***Composite endpoints***	114 (17.4)	42 (50.6)	28 (45.2)	<0.001 *†
30-day in-hospital mortality	5 (0.8)	4 (4.8)	2 (3.2)	0.005 *
Myocardial infarction	5 (0.8)	2 (2.4)	2 (3.2)	0.106
Acute kidney injury	97 (14.8)	36 (43.4)	24 (38.7)	<0.001 *†
Prolonged ventilator care over 24 h	20 (3.1)	6 (7.2)	5 (8.1)	0.037 †
***Postoperative data***				
Length of ICU stay (day)	3 [2, 3]	4 [3, 5]	3 [3, 5]	<0.001 *†
Length of hospital stay (day)	13 [11, 15]	18 [13, 24]	15 [12, 20]	<0.001 *†
***Long-term all-cause mortality***	16 (2.4)	8 (9.6)	7 (11.3)	<0.001 *†
Cardiovascular	6 (37.5)	1 (12.5)	4 (57.1)	
Multi-organ failure	6 (37.5)	7 (87.5)	3 (42.9)	
Unspecified	4 (25.0)	0	0	

Data are presented as median [interquartile range] or n (%). * *p* < 0.05 Euthyroid vs. low T3, † *p* < 0.05 Euthyroid vs. SCH, representing post-hoc analysis. ICU (intensive care unit); SCH (subclinical hypothyroidism); T3 (triiodothyronine).

**Table 4:** Logistic regression analyses of risk factors for the composite endpoints.

	Univariable			Multivariable		
Variables	OR	95% C.I.	*p*-Value	OR	95% C.I.	*p*-Value
Age	1.037	(1.017–1.057)	<0.001	1.028	(1.007–1.049)	0.010
Female	1.620	(1.123–2.338)	0.010			
Body mass index	0.926	(0.881–0.974)	0.003			
Hypertension	1.425	(0.978–2.074)	0.065			
Atrial fibrillation	1.407	(0.629–3.139)	0.407			
CKD	5.534	(3.691–8.297)	<0.001	2.677	(1.671–4.289)	<0.001
CVA	1.667	(1.081–2.569)	0.021			
Diabetes mellitus	1.924	(1.373–2.696)	<0.001			
COPD	1.518	(0.767–3.003)	0.230			
PAOD	1.644	(0.844–3.202)	0.144			
CHF	1.555	(0.876–2.760)	0.131			
Recent MI	1.526	(1.050–2.219)	0.027			
ACS	1.269	(0.910–1.769)	0.161			
Left main disease	1.239	(0.769–1.997)	0.379			
Hypoalbuminemia	3.795	(2.463–5.847)	<0.001			
Anemia	3.354	(2.385–4.715)	<0.001	2.029	(1.375–2.995)	<0.001
EuroSCORE	1.196	(1.119–1.277)	<0.001	1.138	(1.057–1.225)	0.001
Low T3	4.301	(2.682–6.896)	<0.001	2.294	(1.330–3.955)	0.003
SCH	3.458	(2.022–5.913)	<0.001	2.845	(1.582–5.116)	<0.001

ACS (acute coronary syndrome); CHF (congestive heart failure); C.I. (confidence interval); CKD (chronic kidney disease); COPD (chronic pulmonary obstructive disease); CVA (cerebrovascular accident); EuroSCORE (European System for Cardiac Operative Risk Evaluation); MI (myocardial infarction); LM (left main); OR (Odds ratio); PAOD (peripheral artery occlusive disease); SCH (subclinical hypothyroidism); T3 (triiodothyronine).

**Table 5:** Cox regression analyses of risk factors for long-term mortality.

	Univariable			Multivariable		
Variables	HR	95% C.I.	*p*-Value	HR	95% C.I.	*p*-Value
EuroSCORE	1.143	(1.021–1.279)	0.020			
Low T3	4.467	(1.905–110.472)	0.001	3.909	(1.603–19.534)	0.003
SCH	4.774	(1.963–111.608)	0.001	4.807	(1.977–111.690)	0.001

C.I.; confidence interval; HR, hazard ratio; SCH, subclinical hypothyroidism; T3, triiodothyronine.

## Data Availability

The data presented in this study are available on request from the corresponding author. The data are not publicly available due to confidentiality.
